# Supplementary material for: Increased quadriceps intermuscular adipose tissue in chronic liver disease is associated with an altered muscle transcriptome compared with healthy age matched controls
Source: GeroScience. 2025 Nov 14;48(4):6047–61. doi: 10.1007/s11357-025-01982-2 (PMC13575061; doi:10.1007/s11357-025-01982-2)
Supplement: Supplementary file 1 [file 11357_2025_1982_MOESM1_ESM.docx]

|  | **CLD** | **HC** | **P-value** | **Cohen’s d effect size** |
| --- | --- | --- | --- | --- |
| **Quadriceps** |  |  |  |  |
| 20% | 17.2 (4.9) | 10.1 (4.3) | **<0.0001** | 1.01 |
| 40% | 13.0 (3.4) | 7.0 (2.4) | **<0.0001** | 1.30 |
| 50% | 12.0 (3.2) | 6.6 (2.6) | **<0.0001** | 1.20 |
| 60% | 12.4 (3.5) | 6.5 (2.6) | **<0.0001** | 1.22 |
| 80% | 13.8 (4.3) | 6.9 (2.6) | **<0.0001** | 1.23 |
| **Psoas** | 17.3 (5.3) | 10.1 (2.9) | **<0.0001** | 1.77 |

**Supplementary table 1:** IMAT values for the quadriceps and psoas of patients with CLD (CLD) and health control participants (HC) across a range of anatomical locations. For the quadriceps, 20% reflects the most distal point and 80% most proximal point.

|  | **Mean 1 (SD)** | **Mean 2 (SD)** | **P-value** | **Cohen’s d effect size** |
| --- | --- | --- | --- | --- |
| **CLD Quadriceps** |  |  |  |  |
| 20% vs. 40% | 17.25 (4.9) | 13.0 (3.4) | **<0.0001** | 0.64 |
| 20% vs. 50% | 17.25 (4.9) | 12.0 (3.2) | **<0.0001** | 0.8 |
| 20% vs. 60% | 17.25 (4.9) | 12.4 (3.5) | **<0.0001** | 0.72 |
| 20% vs. 80% | 17.25 (4.9) | 13.8 (4.3) | **<0.0001** | 0.48 |
| 40% vs. 50% | 13.0 (3.4) | 12.0 (3.2) | **<0.01** | 0.2 |
| 40% vs. 60% | 13.0 (3.4) | 12.4 (3.5) | NS | 0.11 |
| 40% vs. 80% | 13.0 (3.4) | 13.8 (4.3) | NS | 0.14 |
| 50% vs. 60% | 12.0 (3.2) | 12.4 (3.5) | NS | 0.08 |
| 50% vs. 80% | 12.0 (3.2) | 13.8 (4.3) | **<0.0001** | 0.33 |
| 60% vs. 80% | 12.4 (3.5) | 13.8 (4.3) | **<0.001** | 0.25 |
| **HC Quadriceps** |  |  |  |  |
| 20% vs. 40% | 10.1 (4.3) | 7.0 (2.4) | **<0.001** | 0.56 |
| 20% vs. 50% | 10.1 (4.3) | 6.6 (2.6) | **<0.0001** | 0.62 |
| 20% vs. 60% | 10.1 (4.3) | 6.5 (2.6) | **0.0001** | 0.64 |
| 20% vs. 80% | 10.1 (4.3) | 6.9 (2.6) | **<0.01** | 0.57 |
| 40% vs. 50% | 7.0 (2.4) | 6.6 (2.6) | NS | 0.10 |
| 40% vs. 60% | 7.0 (2.4) | 6.5 (2.6) | NS | 0.13 |
| 40% vs. 80% | 7.0 (2.4) | 6.9 (2.6) | NS | 0.02 |
| 50% vs. 60% | 6.6 (2.6) | 6.5 (2.6) | NS | 0.02 |
| 50% vs. 80% | 6.6 (2.6) | 6.9 (2.6) | NS | 0.07 |
| 60% vs. 80% | 6.5 (2.6) | 6.9 (2.6) | NS | 0.10 |

**Supplementary table 2:** IMAT values for the quadriceps of patients with CLD (CLD) and health control participants (HC) across a range of anatomical locations with 20% being most distal and 80% most proximal.

|  | **CLD** | **HC** | **P-value** | **Cohen’s d effect size** |
| --- | --- | --- | --- | --- |
| **Quadriceps** |  |  |  |  |
| **RF** | 14.1 (3.7) | 6.0 (2.3) | **<0.0001** | 1.67 |
| **VL** | 12.2 (4.0) | 4.9 (2.6) | **<0.0001** | 1.37 |
| **VM** | 11.9 (3.3) | 4.8 (2.0) | **<0.0001** | 1.65 |
| **VI** | 9.3 (3.1) | 4.1 (1.8) | **<0.0001** | 1.30 |
|  |  |  |  |  |
| **CLD** | **Mean 1 (SD)** | **Mean 2 (SD)** |  |  |
| RF vs. VL | 14.1 (3.7) | 12.2 (4.0) | **<0.01** | 0.33 |
| RF vs. VM | 14.1 (3.7) | 11.9 (3.3) | **<0.001** | 0.40 |
| RF vs. VI | 14.1 (3.7) | 9.3 (3.1) | **<0.0001** | 0.90 |
| VL vs. VM | 12.2 (4.0) | 11.9 (3.3) | NS | 0.04 |
| VL vs. VI | 12.2 (4.0) | 9.3 (3.1) | **<0.0001** | 0.51 |
| VM vs. VI | 11.9 (3.3) | 9.3 (3.1) | **<0.0001** | 0.53 |
| **HC** |  |  |  |  |
| RF vs. VL | 6.0 (2.3) | 4.9 (2.6) | NS | 0.31 |
| RF vs. VM | 6.0 (2.3) | 4.8 (2.0) | NS | 0.35 |
| RF vs. VI | 6.0 (2.3) | 4.1 (1.8) | NS (P=0.06) | 0.58 |
| VL vs. VM | 4.9 (2.6) | 4.8 (2.0) | NS | 0.02 |
| VL vs. VI | 4.9 (2.6) | 4.1 (1.8) | NS | 0.21 |
| VM vs. VI | 4.8 (2.0) | 4.1 (1.8) | NS | 0.24 |

**Supplementary table 3:** IMAT values for the Rectus Femoris (RF), Vastus Lateralis (VL), Vastus Medialis (VM) and Vastus Intermedius (VI) of patients with CLD (CLD) and health control participants (HC).
